# Tha cahly project: promoting health literacy about cannabis among the youth

**DOI:** 10.1192/j.eurpsy.2025.581

**Published:** 2025-08-26

**Authors:** B. Serra-Sarró, E. Caballeria, C. Oliveras, P. Guzmán, M. Ballbé, P. Bruguera, D. Ilzarbe, H. López-Pelayo, A. Marta, M. T. Pons-Cabrera, E. Cesari, M. Balcells-Olivero

**Affiliations:** 1 Department of Psychiatry and psychology. Hospital Clínic Barcelona; 2 Health and Addictions Research Group (Grup de Recerca Emergent, 2021 SGR 01158, AGAUR). IDIBAPS. Addictions Unit. Psychiatry and Psychology Service. ICN. Hospital Clinic Barcelona, ICN; 3 Tobacco Control Unit, Cancer Prevention and Control Program, ICO ; Tobacco Control Research Group, Epidemiology, Public Health, Cancer Prevention and Palliative Care Programme, IDIBEL; CIBERES, ISCIII, Madrid, Spain, ICO; 4 Department of Child and Adolescent Psychiatry and Psychology. Hospital Clínic de Barcelona, ICN, Barcelona, Spain

## Abstract

**Introduction:**

The significant psychological burdens posed by cannabis use on adolescents and young adults, make them a target for interventions to enhance their health literacy regarding patterns of cannabis use (dose and frequency) that lead to higher health risks. Critically incorporating this information is known as health literacy (HL). Within the Cahly project we have designed a psychoeducational intervention to enhance HL regarding cannabis use among students aged 15-25 years old.

**Objectives:**

Explore the efficacy of the intervention in improving HL among the students.

**Methods:**

Prospective, randomised controlled trial with a duration of 1-month. 50 high-school centers and universities located in Barcelona were offered to participate. In those accepting, students were randomised to the experimental (received the intervention) or control groups (did not receive it). Both groups completed a baseline questionnaire on: HL, HL on cannabis use, substance use in the previous year and in the previous month, as well as sociodemgraphic data. These questions were repeated after one month of entering the study. In the present stage, we will present the baseline HL level (means, %), and the comparison among groups for the follow-up measures (using either Pearson’s Chi-squared test or Fisher’s exact test as appropriate, group mean comparisons utilized either the Student’s t test or Wilcoxon rank sum test,).

**Results:**

300 students have so far finalised the study. Baseline results indicate low levels of general health literacy, and lower knowledge regarding HL on cannabis, with a predominance of use of informal sources of information. Those receiving the intervention improve significantly their knowledge regarding the patterns of cannabis use that lead to higher risks. However, the intervention did not affect their patterns of substance use.

**Conclusions:**

We propose an innovative intervention aiming to disseminate a definition of cannabis risky use among a vulnerable population such as youth. Preliminary results are promising.

**Disclosure of Interest:**

None Declared

